# Supplementary material for: YAP1 and QSER1 are key modulators of embryonic signaling pathways in the mammalian epiblast
Source: EMBO Rep. 2026 Mar 26;27(9):2369–405. doi: 10.1038/s44319-026-00746-z (PMC13172546; doi:10.1038/s44319-026-00746-z)
Supplement: Supplementary file 8 — Expanded View Figures [file 44319_2026_746_MOESM8_ESM.pdf]

Expanded View Figures

Figure EV1. Controls for *Yap1* cKO embryo analyses.

(**A**) Gel shows expected bands from flox genotyping using a Yap1Flox:cre system. Adapted from Abraham et al, 2025. (**B**) Gels show the genotype of breeding partners. Note that only males carry the Sox2-cre allele to avoid maternal inheritance of Cre activity. (**C**) Same-day genotyping for flox and Cre for fresh-embryo sequencing was performed from the yolk sacs of 14 embryos, simultaneously isolated from 2 pregnant dams. Four controls, indicated in red triangles, and three *Yap1* cKO embryos (floxflox/cre +), shown in blue circles, were pooled and processed for scRNAseq. (**D**) Genotyping of SRY (sex identity) in the 14 embryos isolated for the experimental design of the scRNAseq experiment. (**E**) Violin plot of *Yap1* and *Wwtr1* (TAZ) from scRNAseq expression levels in all clusters comparing *Yap1* cKO to control. *Yap1* expression is significantly reduced in *Yap1* cKO cells across epiblast lineages, including epiblast (adjusted $p = 7.8 \times 10^{-58}$), primitive streak (1.2×10^{-20}), nascent mesoderm (4.2×10^{-17}), cardiac mesoderm (1.2×10^{-5}), blood progenitors, and endoderm (3.8×10^{-4}). Adjusted *p*-values were calculated using a Wilcoxon rank-sum test with Benjamini–Hochberg correction ($*p < 0.05$, $**p < 0.001$, $***p < 0.0001$). Each dot represents a single cell from E7 scRNAseq data. (**F**) Graphs show RT-qPCR analysis of *Yap1* and its target gene, Ccn2 (CTGF), in E7.5 *Yap1* cKO and control embryos ($n = 10$). Data are presented as mean ± SEM. Statistical analysis: Student's t-test, $**p = 0.0072$ and $***p < 0.0008$. (**G**) Graphs display cell cycle S and G2M scores in control and *Yap1* cKO embryos from scRNAseq analysis. Box-and-whisker plots indicate the median (center line), interquartile range (25th–75th percentiles; box), and minimum to maximum values (whiskers). Individual dots represent a single cell from E7 embryo scRNA-seq data. (**H**) Bright-field images of control and *Yap1* cKO E7 embryos. Graphs show cell number quantification per embryo (left) and the size of the epiblast (right) in control and *Yap1* cKO embryos ($n = 8$–10 embryos). Data are presented as mean ± SEM. Statistical analysis: Student's t-test. Scale bar 250 µm. (**I**) Single cell pathway analysis was applied to DEGs. Terms related to TGFb and Wnt signaling pathways significantly enriched (q-value > 1.4, adj. *p*-value < 0.05) in the epiblast are shown. (**J**) Full western blot of nuclear extracts of E7 embryos shown in Fig. 1G. C: control embryos and Y: *Yap1* cKO embryos. Red Arrows indicate bands shown in main Figure; SMAD2/3 (mw: 55 kDa), HISTONE H3 (mw: 15 kDa), GAPDH (mw: 37 kDa), B-CATENIN (mw: 90 kDa). (**K**) Western blot of whole embryo lysates of E7 control and *Yap1* cKO embryos. Pooled embryos numbers are indicated above each lane, along with the makers analyzed and on the right is the full blots. Red Arrows indicate bands that were cropped; SMAD2/3 (mw: 55 kDa), GAPDH (mw: 37 kDa), and B-CATENIN (mw: 90 kDa).

 © The Author(s)

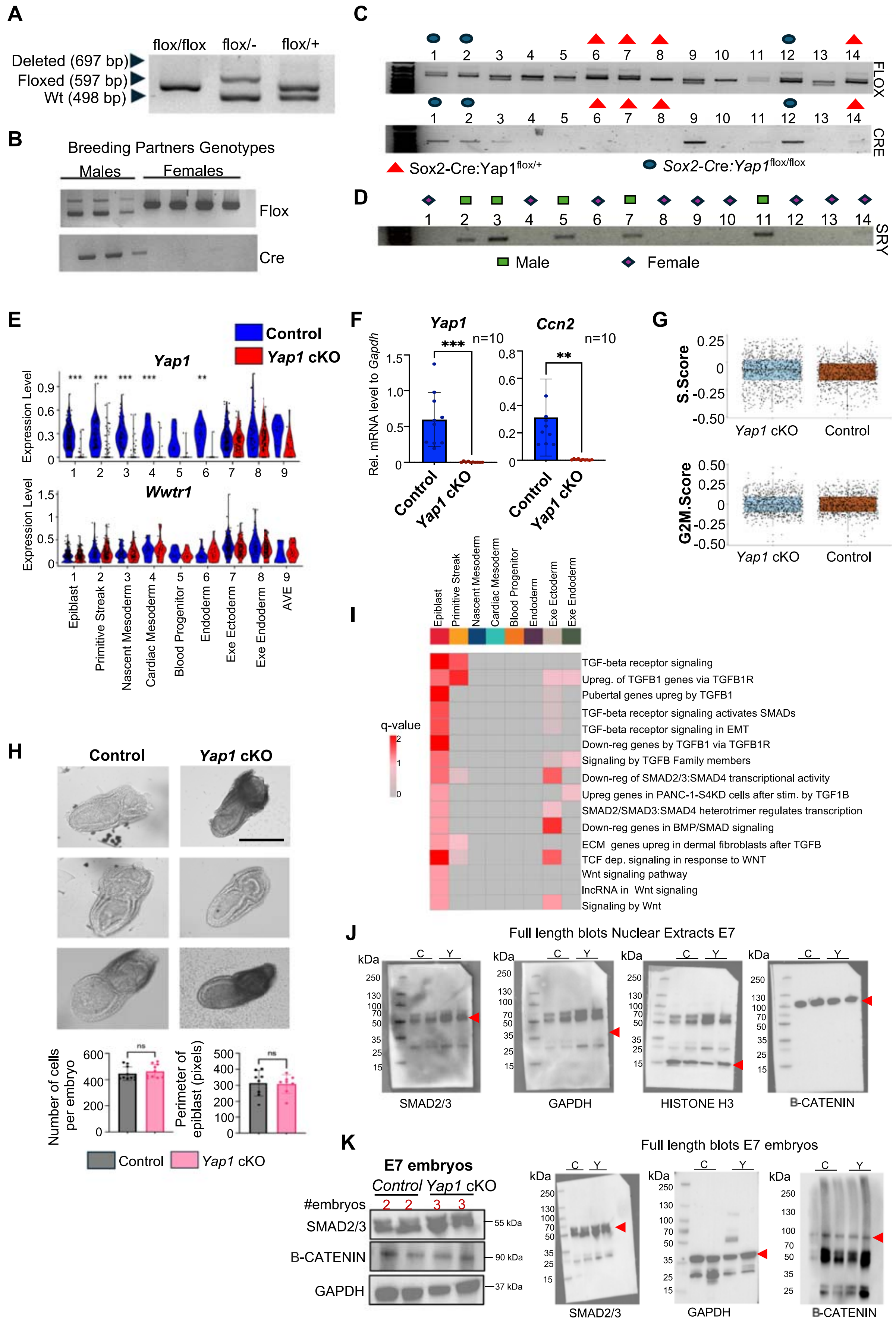
A
flox/flox flox/- flox/+
Deleted (697 bp)
Floxed (597 bp)
Wt (498 bp)
B
Breeding Partners Genotypes
Males Females
Flox
Cre
C
FLOX
CRE
Sox2-Cre:Yap1flox/+
Sox2-Cre:Yap1flox/flox
D
SRY
Male
Female
E
Control
Yap1 cKO
Yap1
Wwtr1
Expression Level
Epiblast
Primitive Streak
Nascent Mesoderm
Cardiac Mesoderm
Blood Progenitor
Endoderm
Exe Ectoderm
Exe Endoderm
AVE
F
Yap1
Ccn2
n=10
Rel. mRNA level to Gapdh
Control
Yap1 cKO
G
S.Score
G2M.Score
Yap1 cKO
Control
H
Control
Yap1 cKO
Number of cells per embryo
Perimeter of epiblast (pixels)
ns
I
q-value
Epiblast
Primitive Streak
Nascent Mesoderm
Cardiac Mesoderm
Blood Progenitor
Endoderm
Exe Ectoderm
Exe Endoderm
TGF-beta receptor signaling
Upreg. of TGFB1 genes via TGFB1R
Pubertal genes upreg by TGFB1
TGF-beta receptor signaling activates SMADs
TGF-beta receptor signaling in EMT
Down-reg genes by TGFB1 via TGFB1R
Signaling by TGFB Family members
Down-reg of SMAD2/3:SMAD4 transcriptional activity
Upreg genes in PANC-1-S4KD cells after stim. by TGF1B
SMAD2/SMAD3:SMAD4 heterotrimer regulates transcription
Down-reg genes in BMP/SMAD signaling
ECM genes upreg in dermal fibroblasts after TGFB
TCF dep. signaling in response to WNT
Wnt signaling pathway
lncRNA in Wnt signaling
Signaling by Wnt
J
Full length blots Nuclear Extracts E7
kDa
C Y
SMAD2/3
GAPDH
HISTONE H3
B-CATENIN
K
E7 embryos
Control Yap1 cKO
#embryos 2 2 3 3
SMAD2/3
B-CATENIN
GAPDH
55 kDa
90 kDa
37 kDa
Full length blots E7 embryos

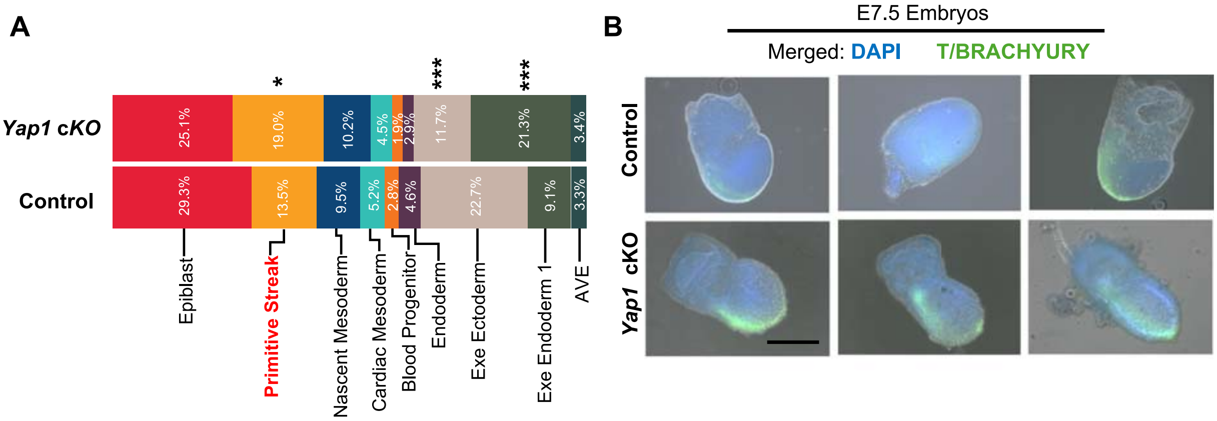

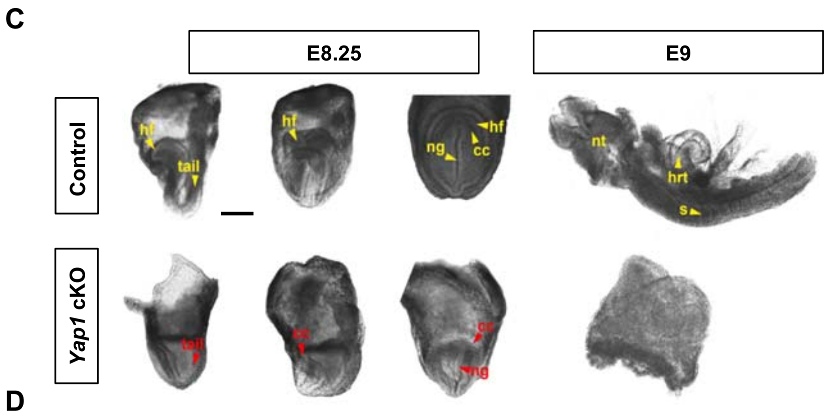

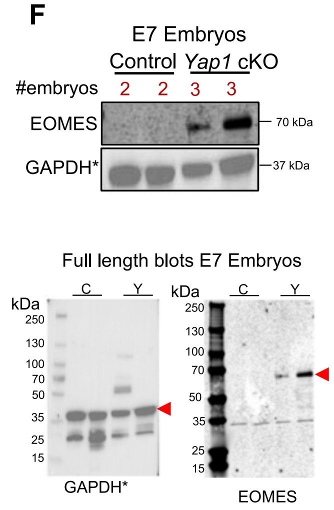

D

E8.25 Phenotyping	Control	*Yap1* cKO
Head folds (hf)	Present (n=15)	Not visible in any of the embryos (n=15)
Cardiac crescent (cc)	Present (n=15)	Visible in 2/3 of the embryos (n=15)
A-P axis	Headfold and tail clearly defined along an A-P axis (n=15)	A-P axis short or not distinguishable (n=15)

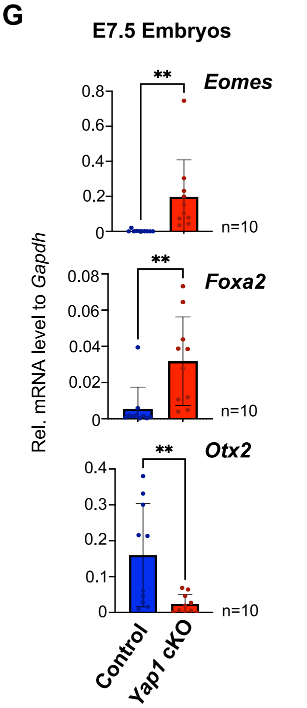

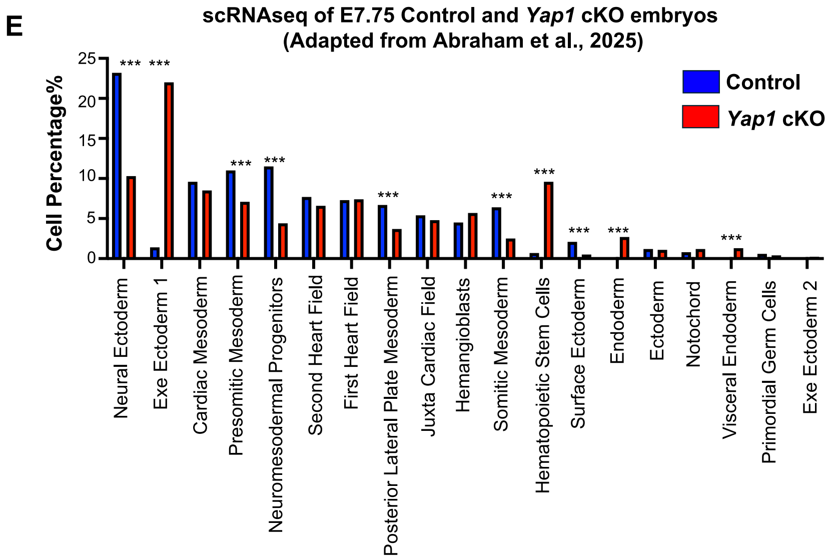

 © The Author(s)

Figure EV2. Phenotyping analysis of *Yap1* cKO embryos reveals patterning defects.

(**A**) Bar graph showing the percentage of cells assigned to each cluster of control and Yap1 cKO embryos. All populations are shown. Statistical analysis: Chi-test, * <0.05, ** <0.001,*** <0.0001. (**B**) Merged images of whole-mount immunostaining of BRACHYURY (T; green) and DAPI (blue) in E7.5 control and *Yap1* cKO embryos. The experiment was repeated three times with different litters with consistent results. See also Fig. 2B. Scale bar 250 µm. (**C**) Bright-field images of control and *Yap1* cKO at E8.25 and E9. hf: head fold, ng: neural groove, cc: cardiac crescent, nt: neural tube, hrt: heart, and s: somites. Scale bar 250 µm. (**D**) Table showing the phenotyping description of control and *Yap1* cKO embryos at E8.25 ($n = 15$). (**E**) Percentage of cells from E7.75 control and *Yap1* cKO scRNA-seq datasets previously published by our lab (Abraham et al, 2025). Clusters marked with an asterisk (*) are significantly different. Statistical analysis: Chi-test with Bonferroni correction run through 100 bootstrap iteration, ***$p < 0.0001$. (**F**) Western blot of whole embryo lysates of control and *Yap1* cKO at E7. Embryos pooled per lane are indicated, along with the markers. The uncropped blots are represented below. The red arrows highlight the molecular weight. C: control Y: *Yap1* cKO Eomes (mw: 70 kDa) and Gapdh (mw: 37 kDa) *Same western blot from Fig. EV1K. (**G**) qPCR of listed markers in E7.5 embryos ($n = 10$). Data are presented as mean ± SEM. Statistical analysis: Student's t-test, **$p = 0.0096$ (Eomes), 0.0068 (Foxa2), and 0.0089 (Otx2).

© The Author(s)

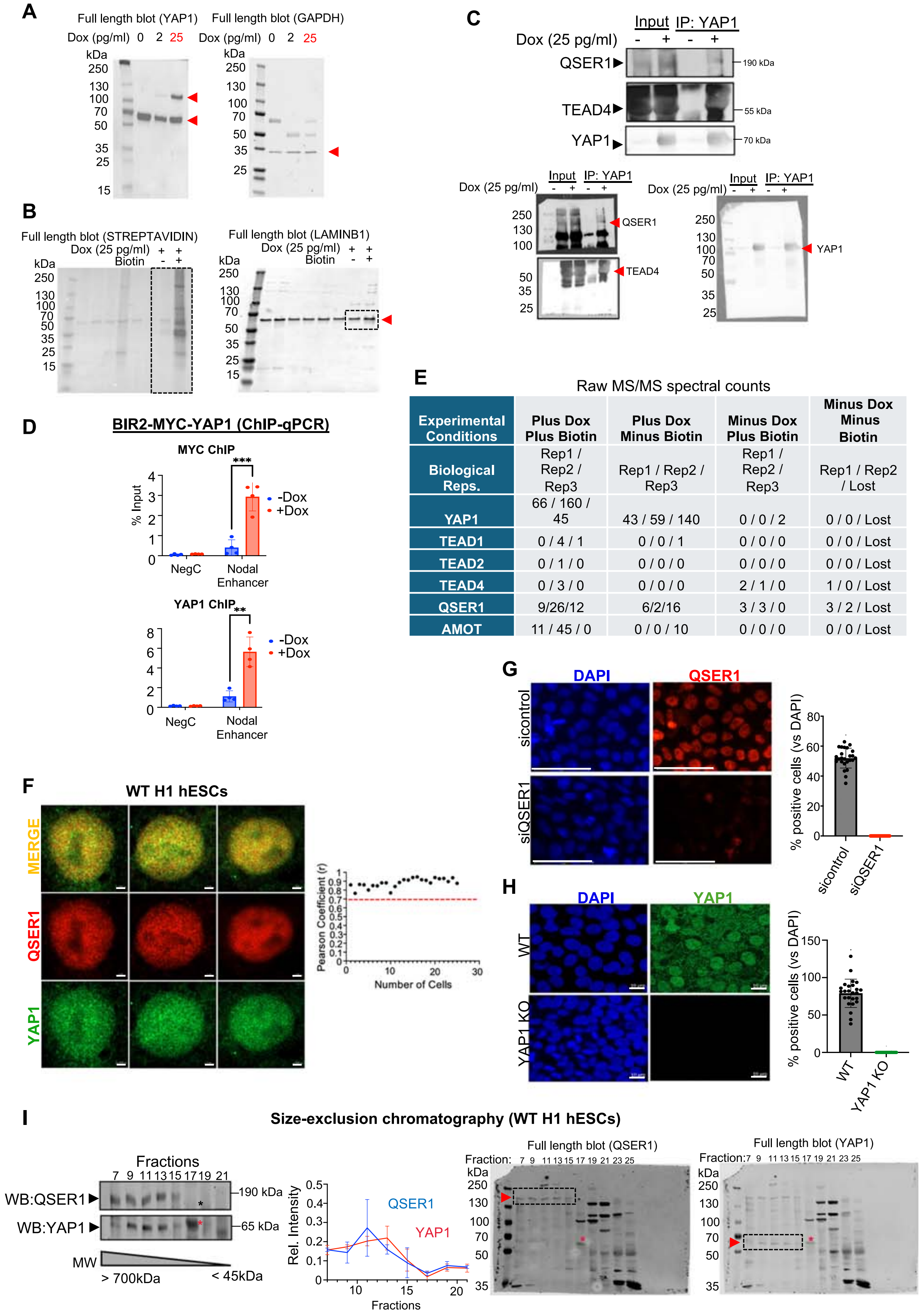

Experimental Conditions	Plus Dox Plus Biotin	Plus Dox Minus Biotin	Minus Dox Plus Biotin	Minus Dox Minus Biotin
Biological Reps.	Rep1 / Rep2 / Rep3	Rep1 / Rep2 / Rep3	Rep1 / Rep2 / Rep3	Rep1 / Rep2 / Lost
YAP1	66 / 160 / 45	43 / 59 / 140	0 / 0 / 2	0 / 0 / Lost
TEAD1	0 / 4 / 1	0 / 0 / 1	0 / 0 / 0	0 / 0 / Lost
TEAD2	0 / 1 / 0	0 / 0 / 0	0 / 0 / 0	0 / 0 / Lost
TEAD4	0 / 3 / 0	0 / 0 / 0	2 / 1 / 0	1 / 0 / Lost
QSER1	9/26/12	6/2/16	3 / 3 / 0	3 / 2 / Lost
AMOT	11 / 45 / 0	0 / 0 / 10	0 / 0 / 0	0 / 0 / Lost

 © The Author(s)

Figure EV3. YAP1 co-localizes with QSER1 in hESCs.

(**A**) Uncropped western blot from Fig. 3B. Red arrowheads indicate bands shown in the main figure. (**B**) Uncropped western blot from Fig. 3C. The dotted square highlights the bands shown in the main figure. (**C**) Co-immunoprecipitation (Co-IP) experiment was performed in the doxycycline-inducible YAP1-Myc-Bir2 clonal hESC line in the presence and absence of Doxycycline, as indicated. The YAP1 antibody was used for immunoprecipitation, and western blot analysis was performed using a QSER1, TEAD4, and YAP1 antibodies. 10% of total lysate was loaded as input. Full western blot shown below. (**D**) ChIP-qPCR analysis was performed in the clonal YAP1-Myc-Bir2 hESC line, in the absence and presence of 25 pg/ml of Doxycycline (Dox). ChIPs using c-myc and YAP1 antibodies were carried out. The genomic regions analyzed are indicated at the bottom. NegC, negative control region ($n = 4$, independent biological replicates). Data are presented as mean ± SEM. Statistical analysis: Student's t-test, $^{**}p = 0.0056$ and $^{***}p = 0.0008$. (**E**) Table depicting experimental conditions used for the BioID2 assay and replicates. Raw MS/MS counts (without normalization) are shown for the indicated proteins in each replicate. Dox: doxycycline. (**F**) Immunostaining of endogenous YAP1 (green) and QSER1 (red) in WT hESCs. Representative 63x magnified images of single cells are shown. Scale bar 2 µm ($n = 25$). On the right, Pearson correlation coefficient (r) was calculated for YAP1 and QSER1 signals in each cell. A threshold of $r > 0.7$ (indicated by the red dashed line) shows a strong positive correlation. (**G**) Immunostaining of QSER1 (red) in sicontrol and siQSER1 in H1 hESCs and quantification of cells positive for QSER1, relative to DAPI (blue). Scale bar 50 µm (25 cells were counted across three images of one biological replicate). Data are presented as mean ± SEM. (**H**) Immunostaining of YAP1 (green) in WT and YAP1 KO H1 hESCs and quantification of cells positive for YAP1, relative to DAPI (blue). Scale bar 10 µm. (25 cells were counted across three images of one biological replicate). Data are presented as mean ± SEM. (**I**) Size-exclusion chromatography was performed on nuclear extracts from WT H1 hESCs. The elution fractions analyzed are indicated on the top. The approximate MW range covered by these fractions is indicated at the bottom. Western blot of QSER1 and YAP1 was performed. Relative intensity was quantified. Complete blots are shown on the right. * shows a band that appeared in both the QSER1 and YAP1 channels and was discarded from quantifications. Experiment was performed with two biological replicates. Data are presented as mean ± SEM.

© The Author(s)

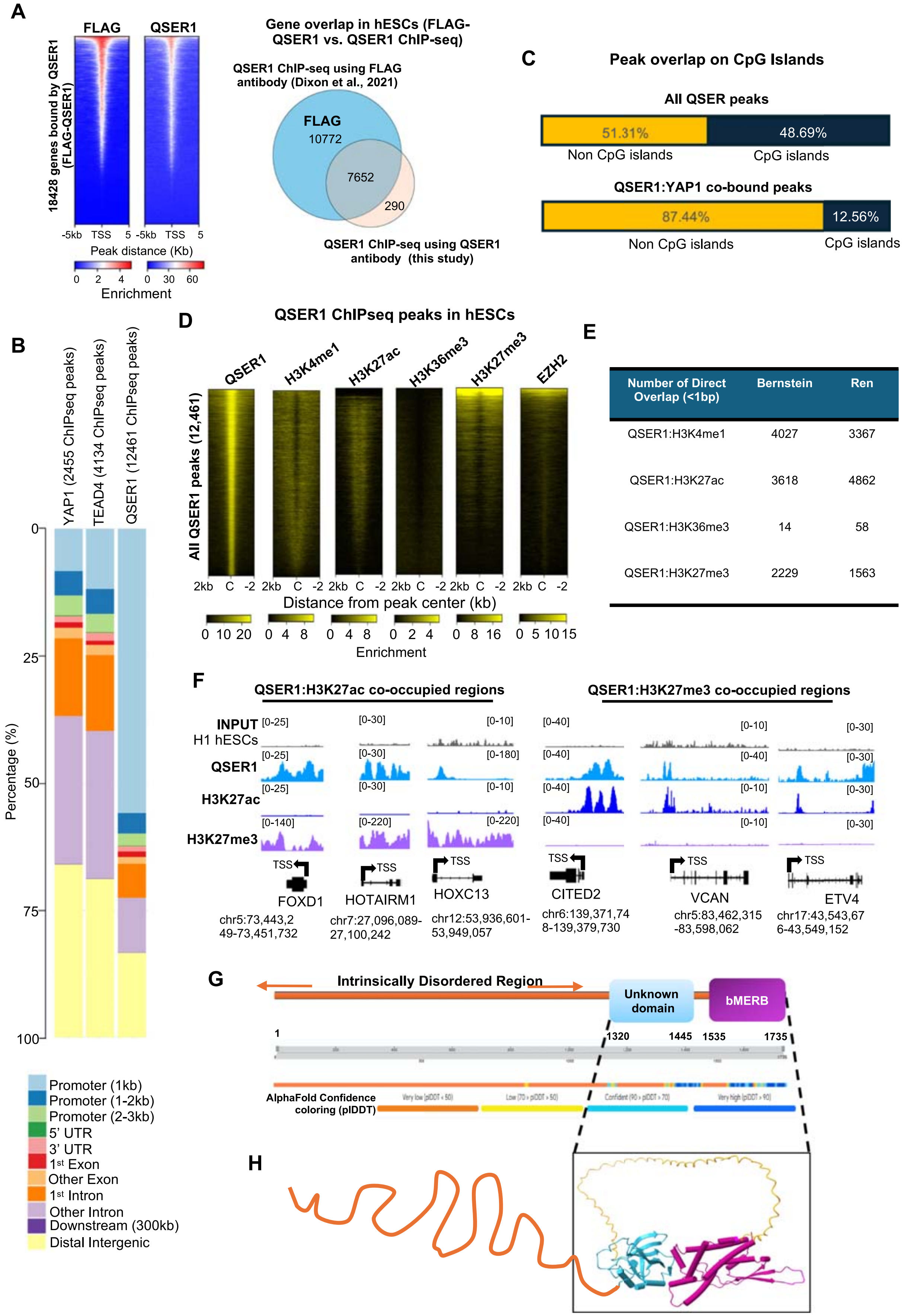
A
FLAG
QSER1
18428 genes bound by QSER1 (FLAG-QSER1)
-5kb TSS 5 -5kb TSS 5
Peak distance (Kb)
0 2 4 0 30 60
Enrichment
Gene overlap in hESCs (FLAG-QSER1 vs. QSER1 ChIP-seq)
QSER1 ChIP-seq using FLAG antibody (Dixon et al., 2021)
FLAG
10772
7652
290
QSER1 ChIP-seq using QSER1 antibody (this study)
C
Peak overlap on CpG Islands
All QSER peaks
51.31%
48.69%
Non CpG islands
CpG islands
QSER1:YAP1 co-bound peaks
87.44%
12.56%
Non CpG islands
CpG islands
B
YAP1 (2455 ChIPseq peaks)
TEAD4 (4134 ChIPseq peaks)
QSER1 (12461 ChIPseq peaks)
Percentage (%)
0
25
50
75
100
Promoter (1kb)
Promoter (1-2kb)
Promoter (2-3kb)
5' UTR
3' UTR
1st Exon
Other Exon
1st Intron
Other Intron
Downstream (300kb)
Distal Intergenic
D
QSER1 ChIPseq peaks in hESCs
QSER1
H3K4me1
H3K27ac
H3K36me3
H3K27me3
EZH2
All QSER1 peaks (12,461)
2kb C -2 2kb C -2 2kb C -2 2kb C -2 2kb C -2 2kb C -2
Distance from peak center (kb)
0 10 20
0 4 8
0 4 8
0 2 4
0 8 16
0 5 10 15
Enrichment
E
Number of Direct Overlap (<1bp) | Bernstein | Ren
QSER1:H3K4me1 | 4027 | 3367
QSER1:H3K27ac | 3618 | 4862
QSER1:H3K36me3 | 14 | 58
QSER1:H3K27me3 | 2229 | 1563
F
QSER1:H3K27ac co-occupied regions
QSER1:H3K27me3 co-occupied regions
INPUT H1 hESCs
QSER1
H3K27ac
H3K27me3
[0-25] [0-30] [0-10] [0-40] [0-10] [0-30]
[0-25] [0-30] [0-180] [0-40] [0-40] [0-30]
[0-25] [0-30] [0-10] [0-40] [0-10] [0-30]
[0-140] [0-220] [0-220] [0-40] [0-10] [0-30]
TSS
FOXD1
chr5:73,443,249-73,451,732
HOTAIRM1
chr7:27,096,089-27,100,242
HOXC13
chr12:53,936,601-53,949,057
CITED2
chr6:139,371,748-139,379,730
VCAN
chr5:83,462,315-83,598,062
ETV4
chr17:43,543,676-43,549,152
G
Intrinsically Disordered Region
Unknown domain
bMERB
1
1320
1445
1535
1735
AlphaFold Confidence coloring (plDDT)
H

◀ **Figure EV4. Genome-wide analysis of QSER1 occupancy in hESCs and protein structure.**

(**A**) Intersection analysis was carried out between our QSER1 ChIP-seq (using QSER1 antibody) and previously published Flag-tagged QSER1 ChIPseq in H1 hESCs (using FLAG antibody) (Dixon et al, 2021). Heatmaps and Venn Diagram show the binding correlation and number of co-bound genes in the two datasets. (**B**) Genomic distribution of QSER1, TEAD4, and YAP1 peaks in WT H1 hESCs from ChIP-seq datasets. (**C**) Graph shows the percentage of QSER1 peaks (all peaks) and QSER1:YAP1 co-bound peaks (199) associated to CpG islands in hESCs. (**D**) Heatmap shows correlation of QSER1 binding, indicated histone marks (source, ENCODE, Bernstein datasets), and EZH2. The heatmap is ranked by QSER1 peak signal and the number of peaks are shown (C = center of the peak, $+/-2$ kb). (**E**) Table shows the number of overlapping QSER1:H3K4me1, QSER1:H3K27ac, QSER1:H3K36me3, and QSER1:H3K27me3 peaks, genome-wide, using two different histone ChIP-seq datasets (Ren and Bernstein). (**F**) IGV genome browser captures show QSER1, H3K27me3, and H3K27ac at indicated genes TSS: transcription start site. (**G**) QSER1 predicted structure based on AF modeling. Most of the protein comprises an intrinsically disordered region (IDR), especially the first three quarters (residues 1 to 1319, orange). Following the large IDR, there are two folded domains. The first is a domain of unknown function (light blue) that is mostly composed of ß-strands, and the second has been designated as a "bivalent Mical/EHBP Rab binding" or bMERB domain (magenta, residues 1535–1735) and is largely helical with three ß-strands in the middle. These two folded domains are separated by 90 residues of a flexible disordered linker (also orange). Below the domain arrangement is a scale bar to denote residue numbers and a bar that represents the AlphaFold2 confidence score (plDDT) with a color key below that shows that darker blue reflects higher confidence. (**H**) Schematic representation of IDR and AlphaFold predictions of QSER1 folded domain structures. Each is color-coded to match the domains shown above. While the relative orientation of the two domains is the most frequently predicted conformation, the linker between them is highly flexible.

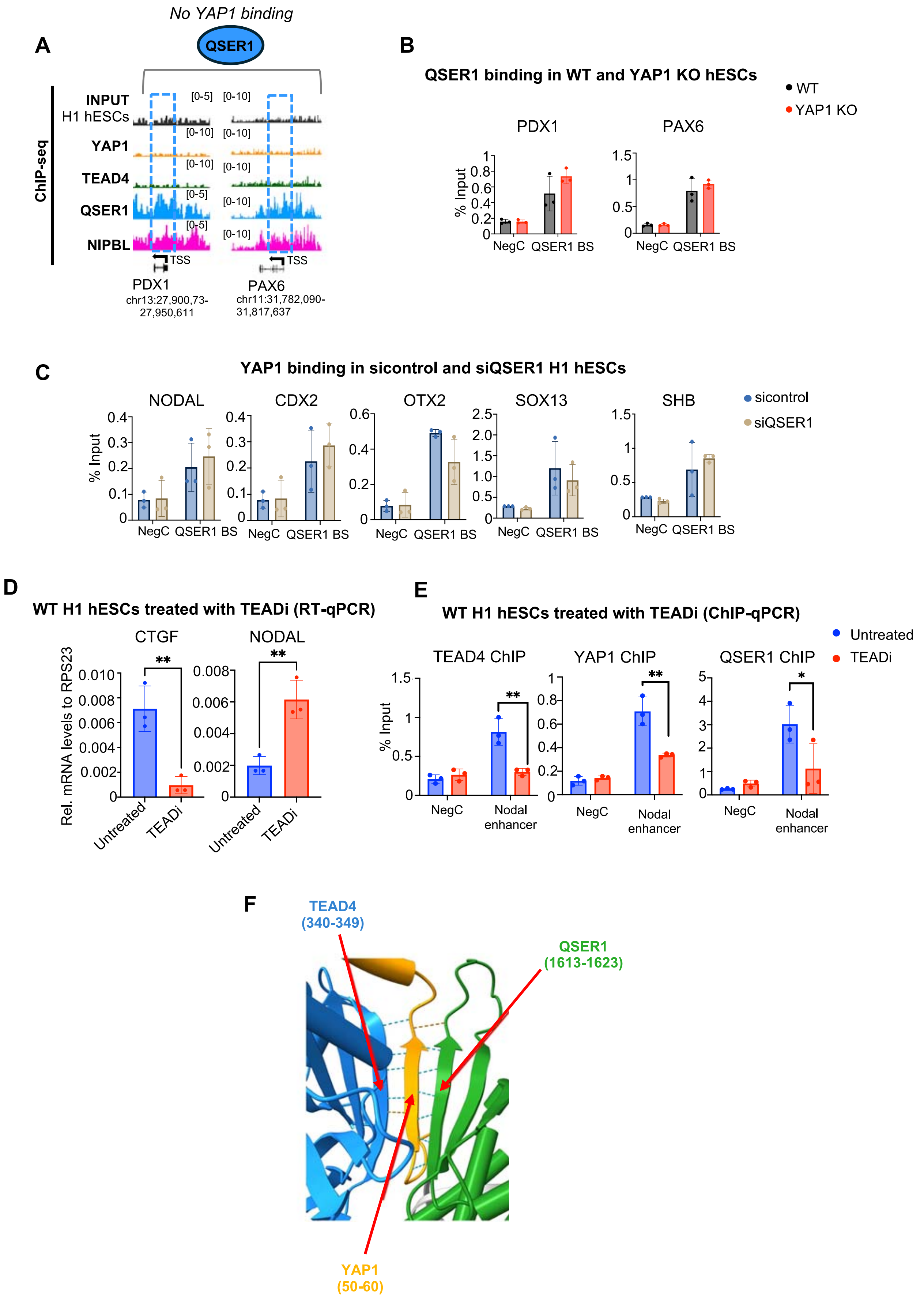
A
No YAP1 binding
QSER1
ChIP-seq
INPUT H1 hESCs
YAP1
TEAD4
QSER1
NIPBL
TSS
PDX1
chr13:27,900,73-27,950,611
PAX6
chr11:31,782,090-31,817,637
B
QSER1 binding in WT and YAP1 KO hESCs
WT
YAP1 KO
PDX1
PAX6
% Input
NegC QSER1 BS
C
YAP1 binding in sicontrol and siQSER1 H1 hESCs
NODAL
CDX2
OTX2
SOX13
SHB
sicontrol
siQSER1
% Input
NegC QSER1 BS
D
WT H1 hESCs treated with TEADi (RT-qPCR)
CTGF
NODAL
Rel. mRNA levels to RPS23
Untreated
TEADi
E
WT H1 hESCs treated with TEADi (ChIP-qPCR)
TEAD4 ChIP
YAP1 ChIP
QSER1 ChIP
Untreated
TEADi
% Input
NegC
Nodal enhancer
F
TEAD4 (340-349)
QSER1 (1613-1623)
YAP1 (50-60)

 © The Author(s)

Figure EV5. QSER1 does not affect YAP1 binding to TEAD4.

(**A**) IGV genome browser snapshots show more examples of distribution of QSER1, YAP1, TEAD4, and NIPBL on indicated genes. (**B**) Graphs show ChIP-qPCR analysis of QSER1 protein on the indicated genomic regions in WT and YAP1 KO hESCs. QSER1 BS: QSER1 binding site. NegC: Negative control region ($n = 3$, independent biological replicates). Data presented as mean ± SEM. Statistical analysis: Student's t-test. (**C**) Graphs show ChIP-qPCR analysis of YAP1 protein on the indicated genomic regions and conditions in sicontrol and siQSER1 conditions. NegC: Negative control region ($n = 3$, independent biological replicates). Data presented as mean ± SEM. Statistical analysis: Student's t-test. (**D**) RT-qPCR of gene expression of CTGF (downstream gene of the Hippo signaling pathway) and NODAL in WT H1 hESCs treated with or without 5 μM GNE-7883 TEAD inhibitor (TEADi) ($n = 3$, independent biological replicates). Data presented as mean ± SEM. Statistical analysis: Student's t-test, $^{**}p = 0.0056$ (CTGF) and $^{**}p = 0.0059$ (NODAL). (**E**) Graph of ChIP-qPCR of TEAD4, YAP1, and QSER1 at enhancer of the NODAL gene in untreated and TEADi treated cells. NegC: Negative control region ($n = 3$, independent biological replicates). Data presented as mean ± SEM. Statistical analysis: Student's t-test, $^{*}p = 0.0148$, $^{**}p = 0.0064$ (YAP1), and $^{**}p = 0.0066$ (TEAD4). (**F**) Molecular modeling of TEAD4 (blue), YAP1 (orange), and QSER1 (green) using AlphaFold3 showing that YAP1 residues 50–60 are tightly bound to QSER1 residues 1613–1623 (7 hydrogen bonds) and TEAD4 residues 340–349 (5 hydrogen bonds, shown as dotted lines). Top ipTM scores for this complex are 0.68, reflecting a high confidence in the conformation of this model.

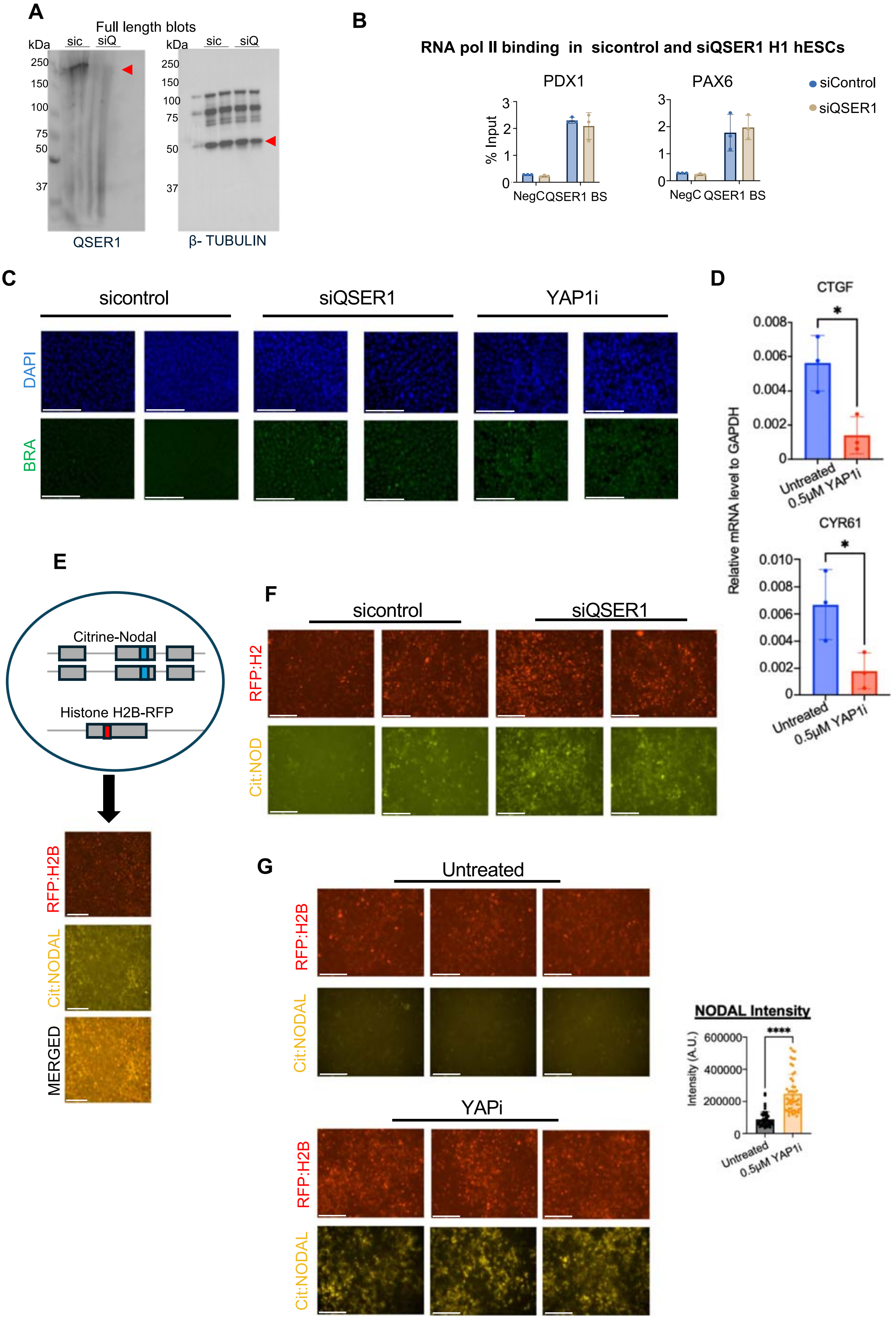
A
Full length blots
kDa
sic
siQ
250
150
100
75
50
37
QSER1
β- TUBULIN
B
RNA pol II binding in sicontrol and siQSER1 H1 hESCs
PDX1
PAX6
siControl
siQSER1
% Input
NegC QSER1 BS
C
sicontrol
siQSER1
YAP1i
DAPI
BRA
D
CTGF
CYR61
Relative mRNA level to GAPDH
Untreated
0.5μM YAP1i
E
Citrine-Nodal
Histone H2B-RFP
RFP:H2B
Cit:NODAL
MERGED
F
RFP:H2
Cit:NOD
G
Untreated
YAPi
NODAL Intensity
Intensity (A.U.)
****

 © The Author(s)

Figure EV6. QSER1 deletion partially phenocopies loss of YAP1 in hESCs.

(**A**) Full uncropped blot of Fig. 6B. Blotted against QSER1 (mw: 190 kDa) and beta-TUBLIN (mw: 50 kDa). Red arrow indicates the band that was cropped. Sic: sicontrol and SiQ: siQSER1. (**B**) Graphs show ChIP-qPCR analysis of RNA polymerase II protein on the indicated genomic regions in sicontrol and siQSER1 hESCs. NegC: Negative control region and QSER1 BS: QSER1 binding site ($n = 3$, independent biological replicates). Data presented as mean ± SEM. Statistical analysis: Student's t-test. (**C**) Additional images of hESC treated with Activin and stained for BRA shown in Fig. 6E. (**D**) Graphs show RT-qPCR analysis of YAP1-target genes CTGF and CYR61 in hESCs untreated and treated with the YAP1 inhibitor (YAPi) DASATINIB for 72 h treatment ($n = 3$, independent biological replicates). Data presented as mean ± SEM. Statistical analysis: Student's t-test, CTGF: $^{*}p = 0.0367$ and CYR61: $^{*}p = 0.0490$. (**E**) Scheme of the Nodal-citrine: H2B-RFP hESC construct with representative fluorescent images of hESCs under basal conditions. (**F**) Additional images of hESC treated with Activin and NODAL shown in Fig. 6G. (**G**) Representative images of untreated and YAP1i treated hESCs treated with Activin (50 ng/mL) for 48 h, NODAL protein expression was visualized using an engineered dual-reporter line expressing NODAL-citrine and H2B-RFP (Liu et al, 2022). Scale bar, 125 μm. Graph shows quantification of fluorescence intensity per cell (50 cells were quantified from three biological replicates). Data presented as mean ± SEM. Statistical analysis: Student's t-test, $^{****}p < 0.0004$.
